# Supplementary material for: In the absence of extensive initial training, cleaner wrasse Labroides dimidiatus fail a transitive inference task
Source: PLoS One. 2023 Jun 23;18(6):e0287402. doi: 10.1371/journal.pone.0287402 (PMC10289426; doi:10.1371/journal.pone.0287402)
Supplement: Attachment — Submitted filename: Response to Reviewers.docx [file pone.0287402.s002.docx]

Response to Reviewer: The manuscript was checked by multiple means to increase the writing quality.

*L38: I suggest rephrasing the final sentence of the abstract:* *Indeed, a parallel study on cleaner wrasse provided positive evidence for TI but was achieved following extensive training on the non-adjacent pairs which may have over-ridden the ecological relevance of the task.*

I agree, the phrasing is better. Thanks.

The manuscript was checked by a correction software then red and corrected again by a native speaker for clarity and grammar.
